# A nuclear ribosomal DNA pseudogene in triatomines opens a new research field of fundamental and applied implications in Chagas disease

**DOI:** 10.1590/0074-02760140398

**Published:** 2015-05

**Authors:** María Angeles Zuriaga, Santiago Mas-Coma, María Dolores Bargues

**Affiliations:** Departamento de Parasitología, Facultad de Farmacia, Universidad de Valencia, Burjassot, Valencia, Spain

**Keywords:** triatomines, Chagas disease, rDNA pseudogene, secondary structures, free energy, functionality

## Abstract

A pseudogene, designated as "ps(5.8S+ITS-2)", paralogous to the 5.8S gene and internal transcribed spacer (ITS)-2 of the nuclear ribosomal DNA (rDNA), has been recently found in many triatomine species distributed throughout North America, Central America and northern South America. Among characteristics used as criteria for pseudogene verification, secondary structures and free energy are highlighted, showing a lower fit between minimum free energy, partition function and centroid structures, although in given cases the fit only appeared to be slightly lower. The unique characteristics of "ps(5.8S+ITS-2)" as a processed or retrotransposed pseudogenic unit of the ghost type are reviewed, with emphasis on its potential functionality compared to the functionality of genes and spacers of the normal rDNA operon. Besides the technical problem of the risk for erroneous sequence results, the usefulness of "ps(5.8S+ITS-2)" for specimen classification, phylogenetic analyses and systematic/taxonomic studies should be highlighted, based on consistence and retention index values, which in pseudogenic sequence trees were higher than in functional sequence trees. Additionally, intraindividual, interpopulational and interspecific differences in pseudogene amount and the fact that it is a pseudogene in the nuclear rDNA suggests a potential relationships with fitness, behaviour and adaptability of triatomine vectors and consequently its potential utility in Chagas disease epidemiology and control.

The genes, basic units for transferring hereditary information, provide structure, function and regulation to a biological system. A gene must go through several steps from a genetic DNA sequence to a fully-functional protein. These steps include transcription, pre-mRNA processing, translation and protein folding. The sequence may be considered nonfunctional if any of the aforementioned steps fails. The most commonly identified disablements are stop codons and frame shifts, which almost universally stop the translation of a functional protein product. Pseudogenes are homologous sequences arising from currently or evolutionarily active genes that have lost their ability to function as a result of disrupted transcription or translation. They may contain stop codons, repetitive elements, have frame shifts and/or lack of transcription. However, they might retain gene-like features. Pseudogenes are of particular interest to biologists since they can interfere with gene centric studies [such as *de novo* gene prediction and polymerase chain reaction (PCR) amplification] and also to evolutionary biologists because of the possibility to study their age and mutational rates and tendencies (Rouchka & Cha 2009).

Although traditionally noted to be nonfunctional, the view of pseudogenes as genetic elements similar to functional genes but without functional properties has recently been put in question (Balakirev & Ayala 2003, Sasidharan & Gerstein 2008). It was traditionally noted that since pseudogenes do not produce protein products, they are typically not under selective evolutionary pressure and thus evolve at rates consistent with neutral drift (Friedberg & Rhoads 2000). Nevertheless, there is recent evidence indicating that a number of pseudogenes may be actively transcribed, as seen in the human and mouse genomes (Zhang et al. 2004 , Harrison et al. 2005, Zheng et al. 2005 ). Although none of these pseudogenes are translated into proteins due to sequence disablements, it has been hypothesised that they may have roles in gene regulation by using their sequences complementary to the homologous functional gene. Transcribed processed pseudogenes have an additional effect that they themselves can become duplicated, resulting in "duplicated-processed" pseudogenes (Zhang & Gerstein 2003, Zhang et al. 2008).

Many characterised pseudogenes have been implicated in regulation of gene expression, gene regulation, and provide a potential source of genetic diversity through recombination with functional genes or exon shuffling (Pavlicek et al. 2006). Interactions have been described between transporter genes and pseudogenes that suggest that expression of the gene is regulated, in part, by transcription of the pseudogene (Piehler et al. 2008). A subset of mammalian pseudogenes has been demonstrated to be responsible for generating small interfering RNAs (siRNAs) by forming double-stranded RNA sequences with the corresponding protein-coding messenger RNA (mRNA) (Tam et al. 2008). These pseudogene-derived siRNAs are then in turn responsible for regulating the functional gene from which the pseudogene originates by acting to degrade the functional gene's mRNA transcripts (Gray et al. 2006, Sasidharan & Gerstein 2008). A classification has even been proposed to differentiate between ghost pseudogenes that have some intermediate functionality (such as a regulatory function or transcriptional activity) and dead pseudogenes that do not have any indication of functionality and therefore are subject to neutral drift (Zheng & Gerstein 2007).

In triatomines vectors of Chagas disease, all pseudogenes so far described have been found in the mitochondrial DNA (mtDNA) genome and proved to be nonfunctional due to the stop codons they present (Mas-Coma & Bargues 2009 ). Recently, however, a new paralogous sequence of the 5.8S gene and the internal transcribed spacer (ITS)-2 of the nuclear ribosomal DNA (rDNA) have been detected to be present in many triatomine species distributed throughout North, Central and northern South America and which has revealed to be a pseudogene with surprising characteristics suggesting potential functionality (Bargues et al. 2014). This appears to be the fourth report of a pseudogene in the nuclear ribosomal operon of an animal, after two in insects such as the grasshopper *Podisma pedestris* (Keller et al. 2006) and the pseudogenic ITS-2 sequences in the malaria vector *Anopheles albitarsis* (Li & Wilkerson 2007 ) and in a vertebrate such as the stone flounder fish *Kareius bicoloratus* (Xu et al. 2009 ).

## Pseudogene verification

**Fig. 1:**
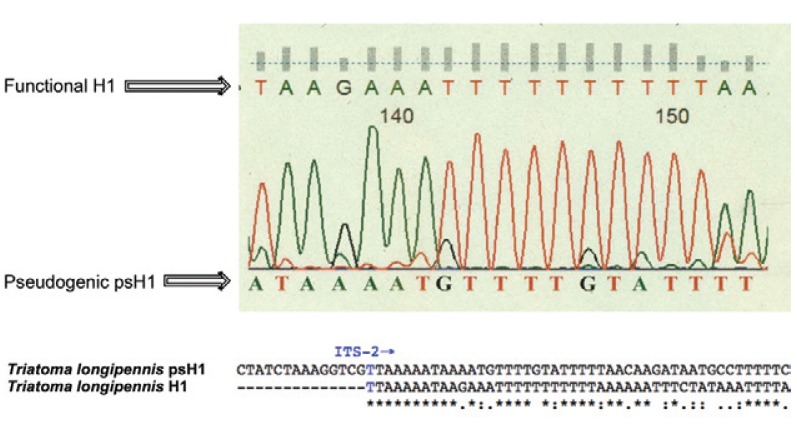
detection of double signal in the sequencing chromatogram fragment obtained when working with a specimen of Triatoma phyllosoma longipennis showing superposition of internal transcribed spacer-2 [functional (H1) and pseudogenic (psH1)]. See corresponding ClustalW2 alignment of the psH1 and H1 paralogous sequences of the haplotype 1 of this species at the bottom.

The coexistence of two different 5.8S+ITS-2 sequences within the same triatomine specimen, one corresponding to the normal functional rDNA operon and another paralogous one corresponding to the pseudogenic, was confirmed by (i) double signal in the sequencing chromatograms (Fig. 1), (ii) cloning and subsequent clone sequencing, (iii) different specific primer sequencing for each functional and paralogous sequences and (iv) relative quantification by real-time PCR (Bargues et al. 2014).

A combination of different criteria was used to identify the pseudogene. In the 5.8S gene, criteria for pseudogene identification included length variability, lower guanine-cytosine content, mutations regarding the functional uniform sequence and relatively high base substitutions in evolutionary conserved sites (Table I). At ITS-2 level, criteria were the shorter sequence and large proportion of insertions and deletions (indels) (Table I) (Bargues et al. 2014).

**TABLE I.** Comparative data (extreme values and means in parentheses) furnishing criteria for the identification of the "ps(5.8S+ITS-2)" pseudogene in triatomine vectors

	Sequences	
Characteristics	Functional^*a*^	Pseudogenic
5.8S gene		
Length (bp)	133^*b*^	134-147 (137)
Guanine-cytosine content (%)	57.14	46.94-50.75 (50.34)
Mutations + indels^*c*^ [n (%)]	-^*b*^	9 (6.08) + 15 (10.13)
Polymorphic sites [n (%)]	-^*b*^	24 (16.22)
Internal transcribed spacer-2		
Length (bp)	490-513 (494.5)	441-479 (455.0)
Proportion of indels^*c*^ (%)	12.36	21.68

a : in species presenting the pseudogene

b : the functional 5.8S gene sequence was identical in all the triatomines studied;

c : indels = insertions and deletions; bp: base pairs.

*DNA secondary structures* - The tendency of complementary strands of DNA to form double helices is well known since long time ago. Single stranded nucleic acid sequences contain many complementary regions that have the potential to form double helices when the molecule folds back on itself. The resulting pattern of double helical stretches interspersed with loops is what is called the secondary structure of an RNA or DNA. Secondary structure elements may in turn be arranged in space to form three-dimensional tertiary structure, leading to additional noncovalent interactions. Tertiary interactions are weaker than secondary structure and, consequently, RNA folding is regarded as a hierarchical process in which secondary structure forms before tertiary structure (Thirumalai et al. 2001). Since formation of tertiary structure usually does not induce changes in secondary structure, the two processes can be described independently.

Functional RNA molecules [transfer RNA, ribosomal RNAs (rRNAs) etc., as opposed to pure coding sequences], usually have characteristic spatial structures and therefore also characteristic secondary structures, that are prerequisites for their function. Their secondary structures are therefore highly conserved in evolution (Hofacker & Stadler 2008 ). Kinetics of RNA secondary structure formation plays an important role in many biological functions. An RNA secondary structure is considered to be locally optimal if there is no lower energy structure that can be obtained by the addition or removal of a single base pair (bp), where energy is defined according to the widely accepted Turner nearest neighbour model (Lorenz & Clote 2011).

*Pseudogenic 5.8S and ITS-2 secondary structures* *and free energy* - They were different from the functional foldings, different one another, showing less negative values for minimum free energy (mfe) and centroid predictions (Table II). A prediction of the secondary structures was made for both functional and paralogous sequences independently for 5.8S and ITS-2. Although the complete sequence was obtained for the functional 5.8S gene, a long fragment (only lacking the 21 bp at the 5' end) was used for secondary structure prediction and free energy evaluation, to be strict in comparisons between functional and pseudogenic sequences. In the case of ITS-2, sequences used for these comparisons were the complete spacer sequences for both functional and pseudogenic paralogues. Associated free energy values were evaluated using the mfe algorithm (Zuker & Stiegler 1981 , Zuker 1989) and the latest free energy rules (Mathews et al. 2004). Fold predictions and bp probabilities were made using the RNAfold program (Hofacker et al. 1994). For comparative purposes, the stability of the secondary structure of the putative pseudogenic sequences vs. that of functional sequences was assessed, for each single sequence, by (i) calculating their mfe structure, (ii) computing their partition function (pf) structure and (iii) analysing the centroid structure, in mountain plots of their positional height values.

**TABLE II.** Minimum free energy (mfe) prediction for the optimal secondary structure and for the thermodynamic ensemble of the functional (FuH) and pseudogenic (PsH) paralogous sequences of the 5.8S ribosomal RNA gene and the ribosomal DNA internal transcribed spacer (ITS)-2 of selected haplotypes representing 13 Triatominae taxa as comparison examples

		mfe prediction		Thermodynamic ensemble prediction			
Species, subspecies and haplotypes	Origin	Optimal secondary structure (kcal/mol)		Free energy thermodynamic ensemble (kcal/mol)	Frequency of the mfe structure (%)	Ensemble diversity	Centroid secondary structure (kcal/mol)
FuH 5.8S							
*Triatoma dimidiata dimidiata* H1^*a*^	Guatemala	-50.30		-52.92	1.42	18.76	-44.80
PsH 5.8S							
*T. d. dimidiata* psH1	Guatemala	-34.90		-37.80	0.90	41.72	-23.50
*T. d. capitata* psH1	Colombia	-35.40		-38.32	0.88	36.44	-25.98
*T. d. maculipennis* psH1	Mexico	-32.90		-35.90	0.78	37.40	-26.91
*T*. sp. aff. *dimidiata* psH3	Yucatán, Mexico	-34.19		-36.68	1.51	36.65	-22.80
*Triatoma phyllosoma phyllosoma* psH1	Mexico	-32.90		-35.97	0.69	40.89	-18.91
*Triatoma mexicana* psH1	Mexico	-32.90		-35.97	0.69	40.89	-18.91
*Triatoma nitida* psH1	Guatemala	-32.90		-35.97	0.69	40.89	-18.91
*Triatoma sanguisuga* psH1	Georgia, USA	-38.20		-41.14	0.84	40.06	-23.30
FuH ITS-2							
*T. d. dimidiata* H1	Guatemala	-124.00		-131.46	0	57.49	-122.50
*T. d. capitata* H11	Colombia	-124.70		-132.18	0	54.49	-123.20
*T. d. maculipennis* H18	Mexico	-124.60		-132.37	0	58.77	-123.00
*T. *sp. aff*. dimidiata *H28	Mexico	-124.70		-129.42	0	99.65	-102.00
*T. p. phyllosoma* H1	Mexico	-124.60		-129.12	0	58.82	-115.70
*T. mexicana* H1	Mexico	-127.00		-134.06	0	48.28	-122.30
*T. nitida* H1	Guatemala	-121.90		-128.39	0	44.34	-119.60
*T. sanguisuga* H1	Georgia, USA	-133.10		-142.41	0	48.80	-127.60
PsH ITS-2							
*T. d. dimidiata* psH1	Guatemala	-85.10		-93.83	0	80.04	-74.00
*T. d. capitata* psH1	Colombia	-87.00		-94.92	0	90.10	-75.70
*T. d. maculipennis* psH1	Mexico	-85.87		-94.86	0	123.97	-57.90
*T*. sp. aff. *dimidiata* psH3	Yucatán, Mexico	-80.10		-89.95	0	126.26	-61.90
*T. p. phyllosoma* psH1	Mexico	-86.70		-95.41	0	93.61	-74.30
*T. mexicana* psH1	Mexico	-86.70		-97.03	0	123.58	-61.00
*T. nitida* psH1	Guatemala	-95.70		-102.95	0	127.57	-79.80
*T. sanguisuga* psH1	Georgia, USA	-80.90		-91.19	0	157.72	-37.85

a : haplotype chosen in representation of the identical FuH 5.8S sequence found in all Triatomini species, subspecies and haplotypes analysed.

The mfe structure of an RNA sequence is the secondary structure that contributes a minimum of free energy. The pf sums all Boltzmann weighted free energies of each secondary structure that is possible given an RNA sequence, thus providing the possibility to calculate base pairing probabilities for each possible pair of bases and the obtaining of an ensemble structure depicting the bp probabilities when pf folding is selected. The centroid structure of an RNA sequence is the secondary structure with minimal bp distance to all other secondary structures in the Boltzmann ensemble [with regard to Boltzmann thermodynamic entropy concepts, see recent reappraisal by Kalinin and Kononogov (2005)]. A mountain plot represents a secondary structure in a plot of height vs. position, where the height m_k_ is given by the number of bp enclosing the base at position k, i.e. loops correspond to plateaus (hairpin loops are peaks) and helices to slopes.

**Fig. 2:**
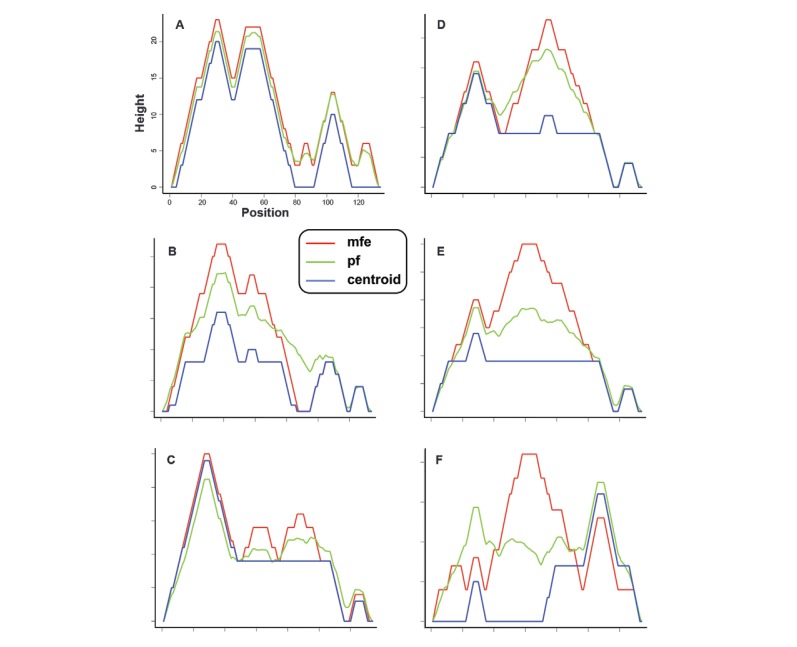
secondary structure mountain plots of height vs. position (height mk = number of base pairs enclosing the base at position k) for the functional and pseudogenic sequences of the 5.8S gene of representative Triatominae taxa: A: functional conserved gene in Triatomini; B: pseudogenic sequence in Triatoma dimidiata dimidiata from Guatemala; C: pseudogenic sequence in T. dimidiata capitata from Colombia; D: pseudogenic sequence in T. sp. aff. dimidiata sensu (Bargues et al. 2008) from Yucatan, Mexico; E: pseudogenic sequence in Triatoma phyllosoma phyllosoma from Mexico; F: pseudogenic sequence in Triatoma sanguisuga from the United States of America. Centroid: centroid structure; mfe: minimum free energy structure; pf: partition function structure.

In the pseudogenic 5.8S gene, the corresponding mfe (delta G) values were less negative, ranging from -32.90 to -38.20 kcal/mol and from -18.91 to -26.91 kcal/mol for optimal and centroid secondary structure prediction, respectively (Table II). The lower fit between the three secondary structure prediction approaches assayed (mfe, pf and centroid) became evident (Fig. 2B-F) when compared with the functional uniform 5.8S secondary structure (Fig. 2A).

**Fig. 3:**
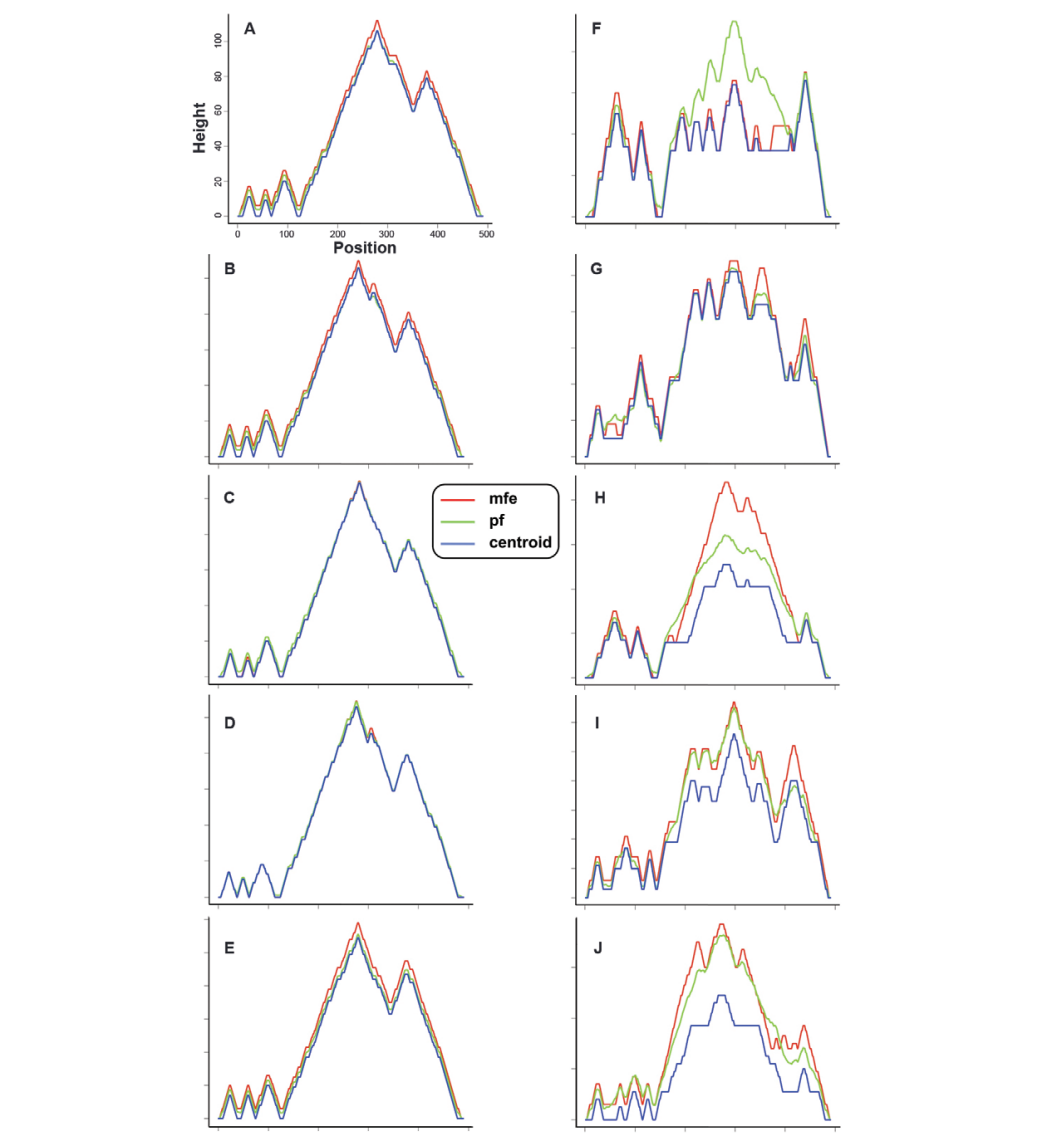
secondary structure mountain plots of height vs. position (height mk = number of base pairs enclosing the base at position k) for the functional and pseudogenic sequences of the internal transcribed spacer-2 of representative Triatominae taxa. Functional sequences (A-E) and pseudogenic sequences (F-J) in Triatoma phyllosoma phyllosoma from Mexico (A, F), Triatoma dimidiata dimidiata from Guatemala (B, G), Triatoma mexicana from Mexico (C, H), Triatoma nitida from Guatemala (D, I), Triatoma sanguisuga from the United States of America (E, J). Centroid: centroid structure; mfe: minimum free energy structure; pf: partition function structure.

At ITS-2, the secondary structures predicted were showing considerably less negative values for mfe and centroid secondary structure predictions (-80.10 to -95.70 kcal/mol and from -37.85 to -79.80 kcal/mol, respectively). Large discrepancy was also observed in the values of ensemble diversity with regard to the same parameters for the functional ITS-2 (Table II). The lower fit between the three secondary structure prediction approaches assayed (mfe, pf and centroid) became very evident (Fig. 3F-J) when compared with their perfect fit in the respective functional ITS-2 secondary structure according to species (Fig. 3A-E), although in given cases the result did not appear bad at all, as in the case of *Triatoma di-* *midiata dimidiata* H1 from Guatemala (Fig. 3G).

## Unique characteristics of the "ps(5.8S+ITS-2)" pseudogene

The combination of several characteristics of the "ps(5.8S+ITS-2)" paralogous sequence detected in triatomine vector species proved it to be a unique pseudogene (Bargues et al. 2014): found in the nuclear DNA instead of in the mtDNA genome; correspond to variants of parts of the nuclear rDNA operon instead of mitochondrial sequences inserted in the nuclear genome (Numts); sequence copies identical inside the same host individual; sequence copies varying inside the same host species; found in an animal, in which such nuclear rDNA pseudogenes appear to be very rare when compared to their detection in plants (Márquez et al. 2003, Manen 2004, Razafimandimbison et al. 2004, Roselló et al. 2007, Thornill et al. 2007, Zheng et al. 2008, Xu et al. 2009, Xiao et al. 2010, Glass et al. 2013); fourth report of a pseudogene in the nuclear ribosomal operon of an animal, after two in insects and one in a vertebrate; first time a pseudogene is found in many different species (13 triatomine taxa) belonging to the same taxonomic group (and not in only 1 species, as in all pseudogenes found in other animal organisms and the majority of those found in plants - only a very few found in a few closely related plant species); not present in all species of Triatominae, but only in given species of the Triatomini tribe; interspecific variability following coherent evolutionary rates; present in phylogenetically closely related triatomine species sharing a geographical distribution, allowing for the assessment of a set of evolutionary characteristics in a pseudogene for the first time.

The discovery of a pseudogene in many phylogenetically related species is unique in animals and allowed for an estimation of its palaeobiogeographical origin based on molecular clock data, inheritance pathways, evolutionary rate and pattern and geographical spread (Bargues et al. 2014).

*Pseudogene distribution* - The presence of both functional and pseudogenic variants of the 5.8S+ITS-2 has been confirmed at least in the following triatomine species (Bargues et al. 2014): *Meccus dimidiata dimidiata*, *M. d. capitata, M. d. maculipennis, M. d. hegneri*, *M*. sp. aff. *dimidiata*, *Meccus phyllosoma phyllosoma*, *M. p. longipennis*, *M. p. pallidipennis*, *M. p. picturata*, *M. p. mazzottii*, *Triatoma mexicana*, *Triatoma nitida *and* Triatoma sanguisuga*.

This pseudogene, paralogous to the functional rDNA 5.8S and ITS-2, has been shown to be absent in the following triatomine species (Bargues et al. 2014): *Triatoma gerstaeckeri*, *Triatoma barberi*, *Triatoma rubida*, *Triatoma ryckmani*, *Triatoma maculata*, *Triatoma infestans*, *Dipetalogaster maxima*, *Panstrongylus megistus* and *Rhodnius prolixus*. The species *Triatoma bassolsae*, in a specimen of which the pseudogene was not found, should be reassessed due to problems posed in the systematic classification of the specimen (Bargues et al. 2014).

This means that the "ps(5.8S+ITS-2)" pseudogene has followed an evolution enabling it to cover a geographical distribution throughout North America, Central America and northern South America [see Carcavallo et al. (1999) regarding the distribution of the triatomine species presenting the pseudogene].

The molecular clock calculations, based on the evolutionary rates in triatomines (Bargues et al. 2000) indicated a relict pseudogene of a very ancient origin with an estimation for the appearance of the pseudogenic sequence of around 11-25.7 million years. Consequently, this additionally suggests that species with older origins or species not related to those in which the pseudogene has been found, should not present the pseudogene. The present geographical distribution of the triatomine taxa presenting the pseudogene suggests, moreover, that the origin of this pseudogene should have taken place in a triatomine ancestor living in the aforementioned old period in Mexico.

*Functionality and applications* - In eukaryotic cells, the vital role of rRNA molecules in protein synthesis leads to strong selection pressure to maintain functional rRNA molecules. The 18S, 5.8S and 28S of the rRNA cistron are produced by a series of nucleolytic reactions that liberate the mature rRNAs from the large primary precursor transcript synthesised by RNA polymerase 1. The three genes are transcribed as a unit by the RNA polymerase 1 into a single precursor pre-rRNA molecule, which undergoes a series of processing steps resulting in mature and fully functional rRNA. These processes include excision of the transcribed spacer regions, nucleotide modifications such as methylation and pseudoridylation, terminal additions of nucleotides and further cleavages and trimming of the precursor molecule (Venema & Tollervey 1999). The excision of the ITS regions is necessary for the production of mature rRNA molecules. Post-transcriptional processes split the cistron, removing two ITS. Thus, ITS-1 and ITS-2 are not incorporated into the structure of the ribosome and are relatively more variable and have less homology. Consequently, ITS-1 and ITS-2 are not subject to the same functional constraints as the rRNA genes and are therefore subject to higher evolutionary rates leading to greater variability in both nucleotide sequence and length.

Spacers play a role in the processing reactions: ITS-1 is required for the processing of the 3' end of the 18S and the 5' end of the 5.8S molecules, while ITS-2 is required for the processing of the 3' end of the 5.8S and the 5' end of the 28S molecules. For this processing of rRNA transcript to be correct and efficient, the proper folding of the ITSs appears to be essential (Côté & Peculis 2001). The importance of ITS secondary structure in rRNA processing is supported by the fact that while the primary sequence of these regions can be quite variable, the secondary structures they form are relatively well conserved across eukaryotes. Even minor modifications to these spacers can inhibit or prevent the formation of mature rRNA products (van Nues et al. 1994).

ITS-2 plays an essential role in the maturation of the pre-RNA as its secondary structures, acquired shortly after transcription, contain the cleavage sites and secondary structure motifs recognised by the enzymatic complexes that act in the processing of the pre-RNA. Factors as biochemical events that disrupt the correct assemblage of its secondary structure have been shown to cause a decrease in the amounts or complete absence of mature 28S rRNA (Hunter et al. 2007). Specific positions within ITS-2 have been identified as sites where processing reactions occur that generate the 7S pre-rRNA and the mature 28S rRNA (Venema & Tollervey 1999). Nucleotides surrounding processing sites are important as substitutions either decrease or eliminate 28S rRNA maturation or lead to decreased cell growth rates. Furthermore, mutations disrupting the secondary structures in the ITS regions may also reduce or eliminate the production of precursor molecules and mature rRNA products (van Nues et al. 1994, Côté & Peculis 2001).

In triatomines, the complete characterisation of the paralogous sequence in question indicated a processed or retrotransposed pseudogenic unit, evolving independently from the concerted evolution acting on the functional normal rDNA operon and following its own concerted evolution. Aspects indicating that this pseudogene follows its own concerted evolution are: (i) quantification demonstrated that there are many copies of the pseudogene inside a triatomine specimen, (ii) cloning allowed to verify that all pseudogene copies have an identical sequence inside the same specimen (no intraindividual pseudogene variability was ever detected), (iii) comparison of different triatomines showed that the pseudogene sequence evolves coherently according to the species phylogeny and (iv) sequence analyses demonstrated that the pseudogene follows an evolutionary rate similar to that of the functional ITS-2 (Bargues et al. 2014). Processed or retrotransposed pseudogenes present distinguishable characteristics indicating RNA processing, including: (i) lack of noncoding intervening sequences (intronic regions and promoters), (ii) presence of poly-A tracts at the carboxyl (3') end and (iii) homological extensions, i.e., flanking repeat regions which are associated with insertion sites of transposable elements (Gibson 1994, Cooper 1999).

The analyses of the triatomine paralogous rDNA sequences also indicated it to be a ghost pseudogene, with some intermediate functionality of the type of a regulatory function or transcriptional activity, at least with regard to the processing of the mature 28S rRNA (Bargues et al. 2014). This would be the case of course if the pseudogene does not expand also including the totality of the large subunit or a part sufficiently long as for truncation and consequent process abolishing. If the pseudogenic ITS-2 would somehow allow for the processing of the mature 28S, this would perhaps be at the origin of changes in behaviour and adaptation capacity, as seen with *T. infestans* ITS-1 minisatellites (Bargues et al. 2006). The fact that divergence differences between functional ITS-2 and pseudogenic ITS-2 did not become higher, despite the long evolutionary period elapsed since the origin of the pseudogene, may suggest that the pseudogene is subject to some constraints instead of evolving free by neutral drift (Bargues et al. 2014).

## Fundamental implications in triatomine research

The existence of two paralogous complete sequences of the ITS-2 inside the same triatomine specimen may be a technical problem easily overlooked in direct sequencing, because the lower stability of the secondary structures of pseudogenic sequences causes them to be preferentially amplified under standard PCR conditions (Harpke & Peterson 2006, Zheng et al. 2008). This may happen when applying somewhat lower annealing temperatures, as widely applied when the same primers are used for a large set of different species. The risk for erroneous sequences represented by the presence of a pseudogenic sequence becomes an important potential problem, given that the ITS-2 is a crucial molecular tool for specimen classification purposes (Mas-Coma & Bargues 2009).

Additional to the aforementioned technical risk to be considered henceforth, this relict pseudogene proves to be a valuable marker for specimen classification, phylogenetic analyses and systematic/taxonomic studies. In the few organisms in which a pseudogene has proved to be relict and to show a substitution rate not outpacing speciation, they have shown great usefulness in reconstructing well supported phylogenetic trees, as verified in pseudogenes with a sequence divergence between them and their corresponding functional copies of about 15% (Zhang et al. 2008). In triatomines, the "ps(5.8S+ITS-2)" pseudogene fulfils the needed requirements: (i) an old origin, (ii) divergences observed between functional sequences and between pseudogenic sequences similar in average and (iii) sufficient resolution (Bargues et al. 2014).

The "ps(5.8S+ITS-2)" pseudogene sequence shows the highest resolution, even at the lowest taxonomic level of subspecies (Bargues et al. 2014): when the same pseudogene was found in specimens presenting different functional ITS-2 haplotype, they proved to belong to the same subspecies; when the same pseudogene was found in different subspecies, in all cases these were subspecies taxa whose validity was already previously put in question by the results obtained in previous functional ITS-2 analyses.

Regarding utility for phylogenetic analyses, the monophyletism of the "ps(5.8S+ITS-2)" pseudogene sequence in a together tree with functional sequences suggests that it no longer interacts genetically with functional copies and thus could be used as another completely new resource to infer phylogeny. Moreover, exhaustive comparative studies made showed that topologies obtained by pseudogenic sequences are more congruent than topologies obtained by functional sequences (Bargues et al. 2014): values of both the consistence index (CI) and the retention index (RI) proved to be very high in the trees reconstructed with pseudogenic sequences, indicating very high congruence of the topologies obtained; for the same paralogous sequences (whether 5.8S+ITS-2 or ITS-2 alone), values of both CI and RI in the trees of the pseudogenic sequences were always higher than those calculated for the trees of the functional sequences; support values for the evaluation of the reliability of the nodes appear to be similar when using the pseudogenic 5.8S+ITS-2 sequence and the functional ITS-2 sequence, sometimes even somewhat higher supports when using the pseudogene.

The comparison of the phylogenetic trees obtained when only using the functional ITS-2 sequence with the tree obtained with the pseudogenic 5.8S+ITS-2 sequence proved that both furnish pronouncedly similar topologies. However, the compared analysis showed some small differences at the level of the nodes which furnished interesting additional information which may be valuable to assess the relationships between species, allowing for the clarification of taxonomic arrangements. The value of the "ps(5.8S+ITS-2)" pseudogene for such purposes should be consequently highlighted. Despite a high indel number, low mutation number and an evolutionary rate similar to the functional ITS-2, that pseudogene distinguishes different taxa and furnishes coherent phylogenetic topologies with resolution similar to the functional ITS-2 (Bargues et al. 2014).

Examples of the taxonomic usefulness of the "ps(5.8S+ITS-2)" pseudogene sequence include (Bargues et al. 2014): (i) the monophyletism it shows for the Phyllosoma complex species and, hence, the support it gives to the validity of the genus *Meccus*, (ii) possibility for a new assessment of the present arrangement of triatomine species in different complexes mainly based on phenotypic characteristics (Schofield & Galvão 2009 ), (iii) confirmation of taxonomic entity at species level, as for instance in the case of *M*. sp. aff. *dimidiata* (Bargues et al. 2008) and (iv) assessment capacity at the level of subspecies, as for instance within the original Phyllosoma subcomplex taxa *M. p. phyllosoma*, *M. p. longipennis*, *M. p. pallidipennis*, *M. p. picturata* and *M. p. mazzottii* (Mas-Coma & Bargues 2009).

## Potential applications for Chagas disease

All data obtained suggest that the "ps(5.8S+ITS-2)" pseudogene should have functionality, as to understand why it has been kept by inheritance throughout triatomine lineages following coherent evolutionary rates and patterns during such a long evolutionary period since its old origin, instead of being lost. Additionally, intraindividual and interpopulational as well as interspecific differences in the amount of the pseudogene, both relative levels and functional ITS-2/pseudogenic ITS ratios assessed by real time PCR, pose a question mark (Bargues et al. 2014). The fact that it is a pseudogene in the nuclear rDNA suggests possible relationships with fitness, behaviour and adaptability of the triatomine vectors, given the crucial role of rRNA molecules in protein synthesis.

It should be considered that nuclear rDNA spacer sequences are noncoding regions typically containing mini and microsatellite repeats (Mas-Coma & Bargues 2009). In that sense, recent studies refer to the involvement of microsatellite polymorphism in the social behaviour of animals (Hammock & Young 2005). Moreover, mini and microsatellites were early suspected to form secondary structures that may play an important role in the mutational process due to their repetitive nature and highly biased nucleotide composition. Expanded repeats in noncoding regions interfere with the metabolism of several cellular pathways, such as methylation, transcription, splicing, RNA processing, nuclear export and translation and the resulting expanded mRNAs often acquire an altered function (Richard et al. 2008).

The transmission of Chagas disease is mainly related to triatomine species adapted to live within human dwellings. An increasing number of species seems to be following a similar adaptive route from sylvatic to domestic habitats (Schofield et al. 1999). The understanding of the capacity of triatomines to colonise the domicile and inherent biological and genetic processes related to this domiciliation process from the wild is of considerable importance in relation to epidemiological surveillance and control of Chagas disease vectors (WHO 1991, Dujardin 1998 ).

DNA techniques are genetic tools which have been used to help in the understanding of the domiciliation capacity of triatomine species (Bargues et al. 2002), not only from *de novo* phenomena in triatomine species nowadays beginning to show a domiciliation trend in different areas, but also in the endeavour of forecasting human habitat re-colonisation by the same previously existing or other sylvatic triatomine species once the effect of insecticide spraying disappears, after control campaigns (Dujardin et al. 2000).

Studies on the main South American vector species *T. infestans* showed a large variation of the haploid DNA content, including a strikingly high DNA content difference between Andean sylvatic populations and non-Andean intradomiciliated specimens (mean reduction of 30%, with a maximum of up to 40%) and a correlation between presence/absence of minisatellites and larger/smaller genome size (Bargues et al. 2006). A relationship between total DNA content per cell, C-heterochromatin revealed by C-banding of chromosomes and highly repetitive DNA sequences has been suggested (Panzera et al. 2004).

The correlation which was observed between the presence or absence of minisatellites in the ITS-1 spacer and genome size is worth mentioning. In Bolivia, both the higher number of minisatellite repeats and the intraspecific variability in the ITS-1 length related to minisatellites agreed with the higher total DNA content and the intraspecific variability of genome size detected in Bolivian populations, which appeared to be larger than the variability found in the numerous populations studied from all other countries (Bargues et al. 2006). Thus, minisatellite repeats in ITS-1 proved to have a parallelism with sylvatic or intradomiciliary populations.

## Concluding remarks

The results of the aforementioned analyses indicate that the "ps(5.8S+ITS-2)" pseudogene constitutes a new valuable marker useful in fundamental and applied studies, for specimen classification, phylogenetic analyses and systematic/taxonomic studies in triatomine vectors, as well as for Chagas disease epidemiology and control, thus opening a new broad research field on the disease throughout North, Central and northern South America.
